# The African Human Microbiome Portal: a public web portal of curated metagenomic metadata

**DOI:** 10.1093/database/baad092

**Published:** 2024-01-10

**Authors:** Anmol Kiran, Mariem Hanachi, Nihad Alsayed, Meriem Fassatoui, Ovokeraye H Oduaran, Imane Allali, Suresh Maslamoney, Ayton Meintjes, Lyndon Zass, Jorge Da Rocha, Rym Kefi, Alia Benkahla, Kais Ghedira, Sumir Panji, Nicola Mulder, Faisal M Fadlelmola, Oussema Souiai

**Affiliations:** Laboratory of Bioinformatics, Biomathematics and Biostatistics (LR16IPT09), Institute Pasteur of Tunis, University Tunis El Manar, Tunis 1002, Tunisia; Faculty of Science of Bizerte, University of Carthage, Tunis, Tunisia; Kush Centre for Genomics and Biomedical Informatics, Biotechnology Perspectives Organization, Khartoum, Sudan; Laboratory of Biomedical Genomics & Oncogenetics, Institut Pasteur de Tunis, University Tunis El Manar, Tunis 1002, Tunisia; The Sydney Brenner Institute for Molecular Bioscience, University of the Witwatersrand, Johannesburg, South Africa; Laboratory of Human Pathologies Biology, Department of Biology, Faculty of Sciences, Mohammed V University in Rabat, Rabat, Morocco; Computational Biology Division, Department of Integrative Biomedical Sciences and Institute of Infectious Disease and Molecular Medicine, Faculty of Health Sciences, University of Cape Town, Cape Town 7925, South Africa; Computational Biology Division, Department of Integrative Biomedical Sciences and Institute of Infectious Disease and Molecular Medicine, Faculty of Health Sciences, University of Cape Town, Cape Town 7925, South Africa; Computational Biology Division, Department of Integrative Biomedical Sciences and Institute of Infectious Disease and Molecular Medicine, Faculty of Health Sciences, University of Cape Town, Cape Town 7925, South Africa; The Sydney Brenner Institute for Molecular Bioscience, University of the Witwatersrand, Johannesburg, South Africa; Laboratory of Biomedical Genomics & Oncogenetics, Institut Pasteur de Tunis, University Tunis El Manar, Tunis 1002, Tunisia; Laboratory of Bioinformatics, Biomathematics and Biostatistics (LR16IPT09), Institute Pasteur of Tunis, University Tunis El Manar, Tunis 1002, Tunisia; Laboratory of Bioinformatics, Biomathematics and Biostatistics (LR16IPT09), Institute Pasteur of Tunis, University Tunis El Manar, Tunis 1002, Tunisia; Computational Biology Division, Department of Integrative Biomedical Sciences and Institute of Infectious Disease and Molecular Medicine, Faculty of Health Sciences, University of Cape Town, Cape Town 7925, South Africa; Computational Biology Division, Department of Integrative Biomedical Sciences and Institute of Infectious Disease and Molecular Medicine, Faculty of Health Sciences, University of Cape Town, Cape Town 7925, South Africa; Kush Centre for Genomics and Biomedical Informatics, Biotechnology Perspectives Organization, Khartoum, Sudan; Laboratory of Bioinformatics, Biomathematics and Biostatistics (LR16IPT09), Institute Pasteur of Tunis, University Tunis El Manar, Tunis 1002, Tunisia; Malawi-Liverpool-Wellcome Trust, Blantyre 3, Malawi; Institute of Infection, Veterinary and Ecological Sciences, University of Liverpool, Liverpool CH64 7TE, UK

## Abstract

There is growing evidence that comprehensive and harmonized metadata are fundamental for effective public data reusability. However, it is often challenging to extract accurate metadata from public repositories. Of particular concern is the metagenomic data related to African individuals, which often omit important information about the particular features of these populations. As part of a collaborative consortium, H3ABioNet, we created a web portal, namely the African Human Microbiome Portal (AHMP), exclusively dedicated to metadata related to African human microbiome samples. Metadata were collected from various public repositories prior to cleaning, curation and harmonization according to a pre-established guideline and using ontology terms. These metadata sets can be accessed at https://microbiome.h3abionet.org/. This web portal is open access and offers an interactive visualization of 14 889 records from 70 bioprojects associated with 72 peer reviewed research articles. It also offers the ability to download harmonized metadata according to the user’s applied filters. The AHMP thereby supports metadata search and retrieve operations, facilitating, thus, access to relevant studies linked to the African Human microbiome.

**Database URL:**  https://microbiome.h3abionet.org/.

## Introduction

The microbiome has been proven to play a pivotal role in the overall health of an individual (1, 2). It is, therefore, understandable that research on the human microbiota has greatly attracted the scientific community’s interest, resulting in the generation of a large number of metagenomic sequencing data. Several high-throughput sequencing data hosting portals, including the European Nucleotide Archive (ENA), the Sequence Read Archive (SRA) and the DNA Data Bank of Japan (DDBJ), have extensively integrated microbiome sequencing data (3–6). The need for a specific set of metagenomic data from public repositories has led to the emergence of targeted databases (DBs), including Metagenomics Rapid Annotation using Subsystem Technology (MG-RAST), Joint Genome Institute Integrated Microbial Genomes and Metagenomes (JGI-IMG/M), MGnify (formerly EBI Metagenomics) and the Global Catalog of Metagenomics (gcMeta) (7–11). Additional specialized resources are related to the environmental microbiome (Marine Metagenomics Portal, Coral Microbiome Database, TerrestrialMetagenomeDB, Animal Microbiome Database [AMDB]), specific to a particular site in the human body (Human Oral Microbiome Database, SKIOME Project, the Human Pan-Microbe Communities [HPMC] database, GMrepo: data repository for Gut Microbiota, mBodyMap) or related to microbiome research analysis (gutMDisorder, MicrobiomeDB, Disbiome database) (12–23).

One of the main aims of these resources is to enable the scientific community to reproduce analyses and benefit from the publicly available data by utilizing them to address new biological research questions. To promote and enhance the reuse of metagenomic data, it’s essential that the descriptions of deposited samples comply with the rigorous Minimum Information about a Metagenomic Sequence (MIMS) standards and FAIR (Findability, Accessibility, Interoperability and Reusability) principles (24–26). Nevertheless, relatively few samples in public repositories comply with the latter criteria. Indeed, the majority of available metadata are incomplete and/or unstructured. Besides, there is a critical lack of common standardized description, for example through the use of ontological terms (27–29).

The above-mentioned inconsistencies are particularly pertinent regarding the African microbiome metadata and its complexities in terms of sociocultural and demographic characteristics (30). Africa has several specificities compared to other continents: it is characterized by the highest levels of human genetic variation and harbors over 3000 different ethnic groups, with more than 2000 different spoken languages (31); yet, data contained in public repositories often have their origin annotated simply as ‘African’. The African continent is also characterized by a wide variety of environments (deserts, plain, tropical rainforests, mountains, savannas, swamps) that directly impact the diet, the subsistence strategies and the susceptibility to diseases of African populations (32) (https://www.ethnologue.com/). Metadata from microbiome studies seldom capture these important features.

Altogether, the aforementioned arguments spurred our interest to develop a dedicated African Human Microbiome Portal (AHMP). The AHMP is a specialized portal of microbiome studies annotated with manually curated, harmonized and standardized microbiome metadata relevant to African populations. It is freely accessible at https://microbiome.h3abionet.org/.

## Materials and methods

**Figure 1.**
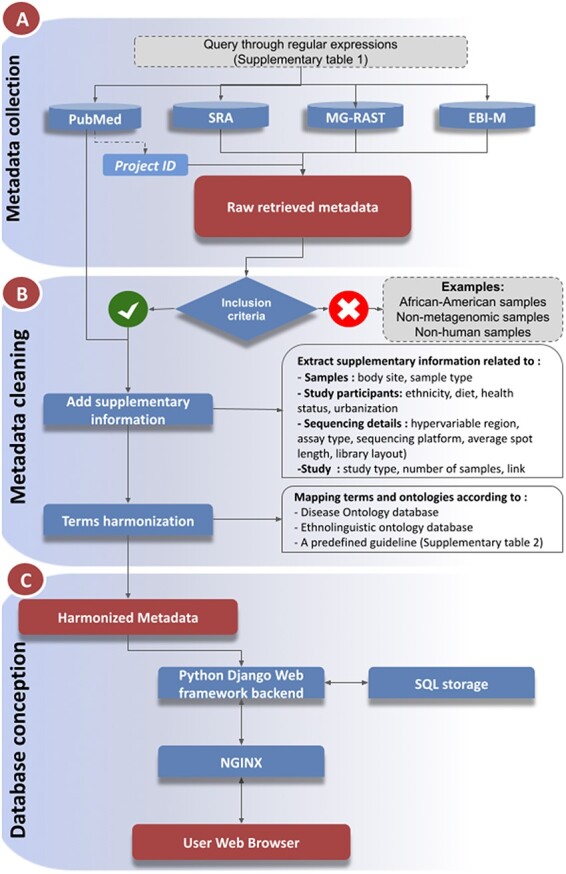
Workflow of metadata collection and database integration. (A): Metadata collection: Metadata were collected from a number of public data repositories. Metadata completion and cleaning (B): The metadata were cleaned, enriched with supplementary information collected from the associated publications, prior to harmonization steps (C): Database and web service constructions.

The database contains metadata of African human metagenomic data from a number of online sequencing data storage resources and publications. The inclusion of information from multiple resources required further data refinement and harmonization, as outlined below and summarized in Figure 1.

Alt text Figure 1: Metadata collection and database integration workflow.

### Metadata search and retrieval

Information on African human metagenomic data was searched and explored from two types of sources: archives and publications, using the keywords provided in Supplementary_TableS1. Metadata for relevant bioprojects and samples were downloaded from corresponding repositories such as Sequence Read Archive (SRA), EBI-Metagenomics (MGnify) and MG-RAST (5, 7, 9, 10) (Figure 1A). We also extended our search to other repositories such as the DDBJ Sequence Read Archive (DRA), Dryad and Integrated Microbial Genomes and Microbiomes Samples (IMG/M) (8, 33). The publication exploration from PubMed redirected us to the associated data and storage repositories. All collected metadata were downloaded in Comma-separated values Comma-separated values (CSV) format.

If a bioproject was not associated with any research publication, it was excluded from the integration. However, excluded projects will be integrated once they are associated with research articles. Only metadata for samples from native populations residing on the African continent were selected (Figure 1B). In addition, non-human and non-metagenomics (Metatranscriptomics, Metametabolomics, etc.) data were excluded. During this curation process, redundant studies and samples were also removed.

### Metadata completion and harmonization

In order to provide comprehensive metadata, we have integrated additional details from the corresponding publications that were not available in the repositories. Such additional information covers participant characteristics (ethnicity, health status, common dietaries, age), locations (urbanization, region), sampling features (body site, sample type), sequencing details (hypervariable region, assay type, sequencing platform) as well as study-related information (study design, number of samples, links to the original publications and to the corresponding data repository) (Figure 1B).

Health status was harmonized by mapping to the Disease Ontology (https://disease-ontology.org/) (34). Similarly, ethnicity information was hyperlinked to the African population ontology AfPO (https://github.com/h3abionet/afpo) (Figure 1B). For the remaining features, harmonization was carried out according to a predefined guideline prior to integration (Supplementary_Table S2).

While harmonizing, we encountered ambiguous values. To overcome such issues, we opted for the following strategy (1): In the absence of geographical coordinates for the samples, the coordinates of the capital were used (2). In some projects, only the year or both month and year of sample collection were provided. If only the year was provided, the first day of the year was assigned as the collection date and the first day of the month in the other case (3). Multiple values are either separated by a semicolon (;) or a double slash (//) for logical operators ‘AND’ and ‘OR’, respectively (4). Missing information was not assigned. Based on the collected information for the repository, a dictionary was generated for data harmonization (supplementary Figure S1). The code used for data correction and harmonization was maintained in Python Jupyter notebooks (https://github.com/h3abionet/african_microbiome_portal) (Supplementary Figure S1).

### Database development

To display the collected and harmonized metagenomic metadata online, we created the web portal accessible at https://microbiome.h3abionet.org/. The portal was developed utilizing open source web technologies, including a Python-Django web-framework for the backend, and Bootstrap v4 for the front-end views, highchartJS for interactive data representation and leafletJS to display sample collection geolocations on the global map (Figure 1C). The source code is available at the repository: https://github.com/h3abionet/african_microbiome_portal. The collected metadata were stored locally in a sqlite3 SQL database (Figure 1C). To integrate the harmonized data to the SQL database, a custom Django command was created to push the CSV information into the SQL tables. An Nginx web server was used to deploy our online portal. A Docker container was also created to host the portal locally (https://github.com/h3abionet/african_microbiome_portal_docker). The source code is available under GNU General Public *License (*GPLv3).

## Results

### Statistical content description

**Figure 2.**
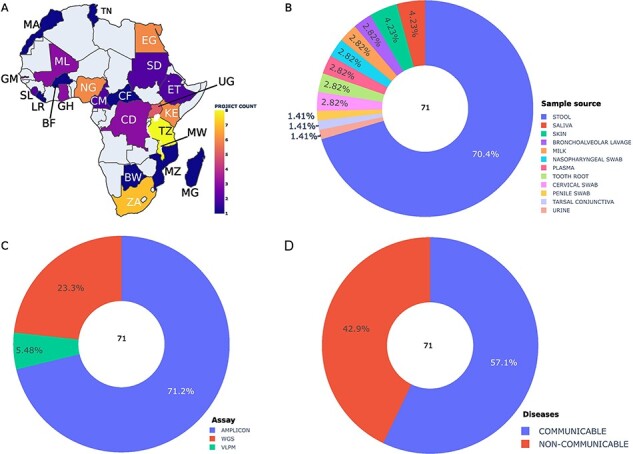
Summary statistics of the collected and integrated metadata by bioprojects. (A) Bioproject distribution according to African countries (A), body site (B), assay type (C) and diseases (D). Abbreviations: BW: Botswana, BF: Burkina Faso, CM: Cameroon, EG: Egypt, ET: Ethiopia, GH: Ghana, KE: Kenya, LE: Liberia, MG: Madagascar, MW: Malawi, ML: Mali, MA: Morocco, MZ: Mozambique, NG: Nigeria, SL: Sierra Leone, ZA: South Africa, UG: Uganda, CF: Central African Republic, CD: Democratic Republic Of The Congo, GM: Gambia, TZ: Tanzania, TN: Tunisia, SD: Sudan.

We collected metadata on 14 899 African human metagenomic samples derived from 70 bioprojects. Sample size per bioproject ranged from a single sample to 3700 samples. The metadata samples originated from 23 African countries, and one bioproject was linked to the Western Sahara region. Malawi (7266 samples), Tanzania (1521 samples), South Africa (1291 samples) and Kenya (919 samples) were the most represented countries in the collected metadata (Supplementary Figure S2A). Metadata related to sub-Saharan populations was more extensively available compared to North African populations (Figure 2A and Supplementary Figure S2). North African populations were limited to Morocco, Tunisia and Egypt (Figure 2A and Supplementary Figure S2A). 83.35% of the collected datasets do not provide information about ethnicity. However, the remaining ones mostly originated from the Hadza ethnic group (56.23%). The gut microbiome was predominantly investigated, representing 85% of the total collected samples, followed by the breast milk and nasopharyngeal metagenome with 3% and 2.87%, respectively (Figure 2B and Supplementary Figure S2B).

Alt text Figure 2: Bioproject distribution based on characteristics.

The most common sequencing platform used in the investigation of African microbiota was Illumina, with 77.80% of all samples. We observed that most African microbiome metagenomic studies used the amplicon sequencing approach, representing 71.2% of bioprojects and 81.8% of samples (Figure 2C and Supplementary Figure S2C). 17.3% of samples and 23.3% of the projects investigated the whole microbial community using Whole Genome Sequencing (WGS) (Figure 2C and Supplementary Figure S2C). Only 5.48% of projects and 0.93% of samples were specifically focused on the viral community through Virus-like particle metagenomics (VLPM) sequencing (Figure 2C and Supplementary Figure S2C).

The data also indicate a higher number of bioprojects related to infectious communicable diseases (CD) compared to non-communicable diseases (NCD) (24 vs 17 bioprojects) (Figure 2D). The projects related to infectious diseases had a particular interest in human immunodeficiency viruses (HIV), diarrhea and bacterial vaginosis, representing particularly 4.54%, 3.25% and 2.52%, respectively, of the collected samples. The NCDs include diseases of anatomical entity (celiac disease), mental health (post-traumatic stress disorder), metabolism (Kwashiorkor, type 2 and type 1 diabetes, environmental enteric dysfunction), immune system disease (atopic dermatitis) and disease of cellular proliferation (colorectal cancer).

### Overview of the functionality of the portal

**Figure 3.**
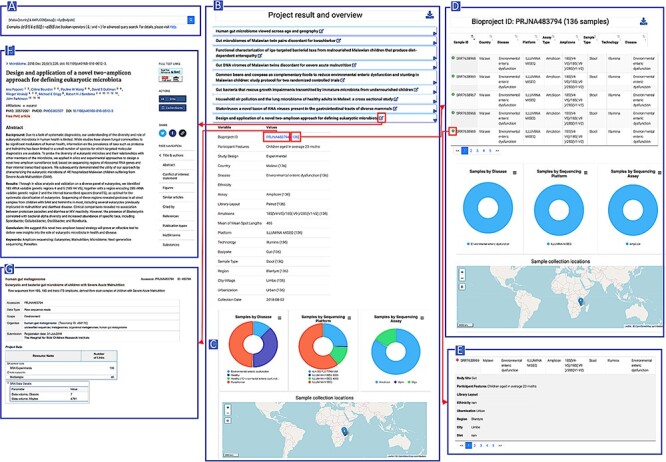
AHMP functionalities: Results can be queried using the search bar on the Home page (A). Example of query results: All the bioprojects related to the query keywords are displayed (B) and results can be further filtered using the interactive charts (D). Minimal information by project (C) or more extended description of samples (E) is also available. A hyperlink to the corresponding publications (F) and the original data (G) is available.

The AHMP homepage provides a textual query interface and a data dashboard. The dashboard allows direct searches based on body site, assay type, country or disease status (Figure 3). The latter query can be conducted either by projects or by samples. Additionally, the interactive African map allows the user to retrieve the projects related to each country. This allows users to visualize country-based statistics and download them in a tabular format.

Alt text Figure 3: African Microbiome Database Portal user interface snapshots.

The textual query (Figure 3A) accepts keywords along with the query type and boolean operators. In the absence of query type, it searches all the fields in database tables for the words queried. Query results are displayed in an accordion-styled table, providing the projects listed publication titles, with general information on the projects displayed when expanded (Figure 3B). The results are downloadable in tabular format. The query results obtained can be further refined using the interactive bar chart and the interactive map (Figure 3D).

Minimal metadata for a selected bioproject such as participant’s features (participant lifestyle, ethnicity, health state, region and city), the samples’ description (sample type) and the sequencing technology used can be displayed as shown by Figure 3C. To retrieve information at sample level, hyperlinks are proposed (Figure 3E). Originating publication and bioproject in public repositories can also be accessed (Figure 3F and G).

### Update and metadata submission into the portal

In addition to regular updates from various repositories, the users and data generators and collectors can submit new or updated African microbiome metadata along with the linked bioproject repositories and samples. To ensure the integration of data in the database, recommendations for minimum metadata fields and format guidelines have been set, and are available in the portal’s help section. The provided data will be checked for errors and harmonized before integration. To facilitate the update and the maintenance of the database, various scripts aiming at automating these processes were implemented. Users can also report issues in the data and repository for its continuous improvements.

## Discussion

African samples are underrepresented in human microbiome studies (30, 31, 35), despite the genetic, ethnic and geographical diversity in the African continent. These data are overshadowed by enormous numbers of data from the global north countries (30). Therefore, it is valuable to highlight and improve annotation of existing African data for better visibility, potential reuse with other studies and meta-analyses. For data reusability, it is critical to have the most complete, accurate and uniform information regarding samples possible, which is often not the case in public repositories (27–29, 36).

A variety of existing tools are available to capture and collect basic metadata, thereby simplifying the access and use of deposited open access data. A recent effort by Kasmanas *et al.* that significantly improved the collection and harmonization of human metagenomic metadata has been implemented in HumanMetagenomeDB (37). As for the remaining resources, they are either not specific to metagenomic metadata (i.e. MetaSRA), limited to a specific human body site microbiota (i.e. GMrepo) or complex to use (20, 29, 38).

Although providing a limited number of African metagenomic studies, the African Human Microbiome Project data portal contains a significant number of attributes (14 889 samples), and most importantly, considers the demographic details of this diverse population. As such, the AHMP offers a better representation of the African human metagenomic metadata, and supports the human microbiome research initiatives in the continent. AHMP also provides access to microbiome analysis tools, training materials and databases consolidating microbiome research.

During metadata collection, we encountered difficulties in locating African metadata in public repositories, even with advanced query keywords, prompting us to extend data searches through the literature. This is probably due to the inadequate annotation and erroneous terms in the public repositories, as stated previously (39, 40). Furthermore, a major investment was required to complete and harmonize the information before integration in the AHMP. Rosenfeld *et al.* (40) reported the predominance of missing information, particularly regarding the age, ethnic group and gender of the participants. This is pertinent when it deals with metadata related to the African populations, which represent different genetic, geographical and environmental backgrounds. Notably, a recent systematic survey of African microbiome studies revealed insufficient consideration of the African ethnicity of the study participants (41). Similarly, we have found a relatively limited number of ethnic groups, mostly centered on the Hadza. Therefore, remaining ethnic groups also need a balanced attention. Likewise, urbanization and the progressive adoption of a Western lifestyle should also be addressed (41). Another noteworthy discernment depicted from the collected metadata is that the microbial community profiling was mostly limited to sub-Saharan populations, in line with the previously raised underrepresentation of North Africans (31).

## Conclusion

In this study, we introduce AHMP, a public web portal that supports tailored metadata specific for the microbiome of the African populations. With its user-friendly interactive interface and query result summaries, this portal aims to provide researchers with carefully curated resource for studies with samples of African origin without requiring them to undergo the laborious process of searching through numerous repositories. The implementation of this database is also a further application of the FAIR principles, since the portal addresses the current need for correctly described metadata, enabling available data to be accessed and interpreted or repurposed to address new biological questions.

## Future steps

Considering that data repositories are essential resources for research conceptualization and reproducibility, a portal must be sustainable for a longer period and evolve feature and data wise over time. Thus, we intend to work towards enriching the portal by incorporating recently published and relevant African human microbiome studies as well as metadata. For this purpose, we have set up a notification system from NCBI and Pubmed. However, there is still an imperative need to sensitize data depositors to the necessity of submitting sufficiently descriptive metadata. The portal’s evaluation is an ongoing process, and it will continue even after it is made publically available to the biomedical research community.

## Supplementary Material

baad092_Supp
