# Supplementary material for: The African Human Microbiome Portal: a public web portal of curated metagenomic metadata
Source: Database (Oxford). 2024 Jan 10;2024:baad092. doi: 10.1093/database/baad092 (PMC10782148; doi:10.1093/database/baad092)
Supplement: baad092_Supp [file baad092_supp.zip › suppl_data/Supplementary Table2.docx]

| **Attributes** | **Description** | **Possible values** | Mandatory/optional | **Harmonization** |
| --- | --- | --- | --- | --- |
| **REPOSITORY ID** | Project ID where the sequences are stored in public repository | usually start with PRJNA or PRJEB or mgp | M | - |
| **REPOSITORY LINK** | Link to Project ID in public repository | link https | M | - |
| **SAMPLE NUMBER** | Number of african samples avaible in public repository | - | M | - |
| **STUDY TITLE** | Title of the publications if published | - | O | - |
| **STUDY LINK** | Link to the publications if published ( Pubmed ID) | PMID (link https) | O | - |
| **ASSAY TYPE** | Assay protocol performed | Amplicon | M | Amplicon |
|  |  | Whole Genome Shotgun |  | WGS |
|  |  | VLP*** |  | VLPM |
| **TECHNOLOGY** | sequencing technology | Illumina | M | Illumina |
|  |  | SOLiD |  | Solid |
|  |  | Roche 454 (formerly Life Sciences) |  | Roche |
|  |  | IonTorrent |  | Ion torrent |
|  |  | PacBio |  | Pacbio |
|  |  | MinION Oxford Nanopore |  | Minion |
| **COUNTRY** | patients' country | - | M |  |
| **DISEASE** | Health status of patients | - | M | i.e (Healthy, Intestinal amoebiasis, HIV, etc) |
| **DOID** | Disease Ontology collected from https://disease-ontology.org/ |  | M |  |
|  |  |  |  |  |
| **STUDY DESIGN** | Type of study | Case-control | M | Case-control |
|  |  | cohort study |  | Cohort study |
|  |  | clinical trial |  | Clinical trial |
|  |  | cross sectional |  | Cross-sectional |
|  |  | descriptive study |  | Descriptive study |
|  |  | Meta analysis |  | Meta-analysis |
|  |  | longitudinal |  | Longitudinal |
|  |  | experimental |  | Experimental |
| **BODY SITE** | The part of the body from which the sample was collected | human gut metagenome | M | Gut |
|  |  | human oral metagenome |  | Oral |
|  |  | human_penil/vaginal/cervicalmetagenome |  | Genital |
|  |  | plasma/ blood metagenome |  | Blood |
|  |  | urine metagenome |  | Urine |
|  |  | human milk metagenome |  | Milk |
|  |  | human eye metagenome |  | Eye |
|  |  | human lung metagenome |  | Lung |
|  |  | human skin metagenome |  | Skin |
|  |  | human nasopharyngeal metagenome |  | Nasopharynx |
| **PARTICIPANT FEATURES** | Additional informations on patints (Age, sex, etc.) | - | O | - |
| **AVERAGE SPOTLENGTH** | read length | - | M | - |
| **SAMPLE NAME** | Sample name as stored in public repository | - | M | - |
| **run ID** |  |  | M |  |
| **Sample ID** | Sample ID as stored in public repository |  | M |  |
| **COLLECTION DATE** | Date of sample collection /Year of collection | - | O | MM-DD-YYYY or YYYY |
| **LIBRARY LAYOUT** |  | Paired-end | M | paired |
|  |  | Single-end |  | single |
| **LON LAT** | Geographical coordinates of the city/country | - | M | Latitude,Longitude of the capital (i.e : -15.7862543,35.0035694) |
|  |  |  |  | (Check can be performed using this website : https://microreact.org/project/) |
| **SAMPLE TYPE** | Type of sample | Human milk | M | Milk |
|  |  | saliva |  | Saliva |
|  |  |  |  | Tooth root/Gingiva |
|  |  |  |  | nose_swab |
|  |  | Human stool, Faecal, fecal sample, gut, stool, fecal sample, gut,Feces |  | stool |
|  |  | Human cervical swab |  | Cervical swab |
|  |  | Urine |  | Urine |
|  |  | tears |  | Tears, Tarsal conjunctiva |
|  |  | paperpoint and sterile file |  |  |
|  |  | Coronal sulcus swab eluent |  | Penile swab |
|  |  | Skin |  | Skin |
|  |  | Bronchoalveolar Lavage |  | Bronchoalveolar lavage |
|  |  | plasma |  | Plasma |
| **ETHNICITY** | Ethinicty of the participants if available | - | O | i.e (Hadza, Bantu,etc) |
| **URBANIZATION** | patient/individual living area | Refugee camp | O | Refugee camp |
|  |  | Urban |  | urban |
|  |  | Peri-urban |  | Peri-urban |
|  |  | rural |  | rural |
| **REGION** | - | - | O | - |
| **CITY/Village** | - | - | O | - |
| **TARGET AMPLICON** | Hypervariable region targated in case of amplicon sequencing | - | M | i.e (v3-v4, v5) |
| **DIET** | Informations on patient/individual dietary regime | - | O | i.e : Mediterranean , Hunter Gatherers Agropastoralist Diet , Ready-to-use therapeutic food |
| **PLATFORM** | sequencing instrument | 454 GS | O | 454 GS |
|  |  | 454 GS 20 |  | 454 GS 20 |
|  |  | 454 GS FLX |  | 454 GS-FLX |
|  |  | 454 GS FLX+ |  | 454 GS-FLX+ |
|  |  | 454 GS FLX Titanium |  | 454 GS-FLX Titanium |
|  |  | 454 GS Junior |  | 454 GS-Junior |
|  |  | Illumina HiSeq |  | Illumina HiSeq |
|  |  | Illumina HiSeq 1000 |  | Illumina Hiseq 1000 |
|  |  | Illumina HiSeq 1500 |  | Illumina Hiseq 1500 |
|  |  | Illumina HiSeq 2000 |  | Illumina Hiseq 2000 |
|  |  | Illumina HiSeq 2500 |  | Illumina Hiseq 2500 |
|  |  | Illumina HiSeq 3000 |  | Illumina Hiseq 3000 |
|  |  | Illumina HiSeq 4000 |  | Illumina Hiseq 4000 |
|  |  | Illumina NovaSeq 6000 |  | Illumina Novaseq 6000 |
|  |  | Illumina MiSeq |  | Illumina Miseq |
|  |  | Illumina MiniSeq |  | Illumina Miniseq |
|  |  | Illumina iSeq 100 |  | Illumina iseq 100 |
|  |  | Illumina HiScanSQ |  | Illumina HiscanSQ |
|  |  | HiSeq X Five |  | Illumina Hiseq X Five |
|  |  | HiSeq X Ten |  | Illumina Hiseq X Ten |
|  |  | NextSeq 500 |  | Illumina NextSeq 500 |
|  |  | MinION |  | MinION |
|  |  | GridION |  | GridION |
|  |  | PromethION |  | PromethION |
|  |  | PacBio RS |  | PacBio RS |
|  |  | PacBio RS II |  | PacBio RS II |
|  |  | Ion Torrent PGM |  | Ion Torrent PGM |
|  |  | Ion Torrent Proton |  | Ion Torrent Proton |
|  |  | Ion Torrent S5 |  | Ion Torrent S5 |
|  |  | Ion Torrent S5 XL |  | Ion Torrent S5 XL |
|  |  | Illumina Genome Analyzer IIx |  | Illumina Genome Analyzer IIx |

***-

Additional guideline:

1. Multiple value are either separated by a semicolon (value 1 ; Value 2) by a double slash ( value 1 // value 2 )
2. Replace the missing metadata by an empty cells (not space)
3. All values starts with a capital letter
4. Composite terms separated by space (i.e South Africa)
