# Supplementary material for: The African Human Microbiome Portal: a public web portal of curated metagenomic metadata
Source: Database (Oxford). 2024 Jan 10;2024:baad092. doi: 10.1093/database/baad092 (PMC10782148; doi:10.1093/database/baad092)
Supplement: baad092_Supp [file baad092_supp.zip › suppl_data/Supplementary_table1.docx]

| **Repository** | **Keywords** | |
| --- | --- | --- |
| **SRA and PubMed** | **Basis Keywords queries:** | ((((Africa) OR african)) AND ((("metagenomic") OR "metatranscriptomic") NOT "genomic")) AND ((((((((("microbiome") OR "microbiota") OR "microflora") OR "16s") OR "18s") OR "phages") OR "virome") OR "mycobiome") OR "metabolome") |
|  | **Advanced Keywords queries** | (((Algeria OR Angola OR Benin OR Botswana OR BurkinaFaso OR “Burkina Faso” OR upperVolta OR "Upper Volta" OR Burundi OR Cameroon OR CapeVerde OR "Cape Vedre" OR Central African Republic OR Chad OR Tchad OR Comoros OR “les comores” OR “IesComores” OR Comoro Islands OR “Comro Islands” OR Congo OR DemocraticrepublicCongo OR “Democratic Republic of the Congo ” OR Zaire OR Djibouti OR Egypt OR Equatorial Guinea OR " Equatorial Guinea " OR Eritrea OR Ethiopia OR Gabon OR Gambia OR Ghana OR Guinea OR Guinea Bissau OR " Guinea Bissau " OR IvoryCoast OR “Ivory Coast” OR Coted'Ivoire OR "Cote d'Ivoire" OR Kenya OR Lesotho OR Liberia OR Libya OR Lbiia OR Jamahiriya OR Jamahiriya OR Madagascar OR Malawi OR Mali OR Mauritania OR Mauritius s OR IleMaurice OR “Ile Maurice” OR Morocco OR Mozambique OR Mozambique OR Namibia OR Niger OR Nigeria OR Rwanda OR SaoTome OR "Sao Tome" OR Senegal OR Seychelles OR Sierra Leone OR "Sierra Leone" OR Somalia OR SouthAfrica OR "South Africa" OR Sudan OR South Sudan OR “South Sudan” OR Swaziland OR Tanzania OR Tanganyika OR Zanzibar OR Togo OR Tunisia OR Uganda OR Western Sahara OR "Western Sahara" OR Zambia OR Zimbabwe OR Africa OR Africa OR Southern Africa OR West Africa OR Western Africa OR Eastern Africa OR East Africa OR North Africa OR Northern Africa OR Central Africa OR SubSaharan Africa OR “ Sub-Saharan Africa” **OR bantu OR "hunter gather" OR pastoralists OR hadza OR pygmy**))  AND (microflora OR microbiota OR microbiome OR metagenome OR metagenomics OR resistome OR phageome OR viromics OR virome OR mycobiom**e NOT metabolomics NOT metabolome NOT metaproteomics NOT metatranscriptomic**)  AND (humans OR human OR HomoSapiens OR « Homo Sapiens”) |
| **MG-RAST** |  | The search was made using countries’ name: Tchad, South Africa, Nigeria, Malawi, Tanzania, Uganda, Sudan, Rwanda, Congo, Cameroon, Botswana, Morocco, Kenya, Mali, Niger |
| **EBI-Metagenomic & DDBJ** |  | **General query terms : African microbiome** |
