# Supplementary figures and images for: The African Human Microbiome Portal: a public web portal of curated metagenomic metadata
Source: Database (Oxford). 2024 Jan 10;2024:baad092. doi: 10.1093/database/baad092 (PMC10782148; doi:10.1093/database/baad092)

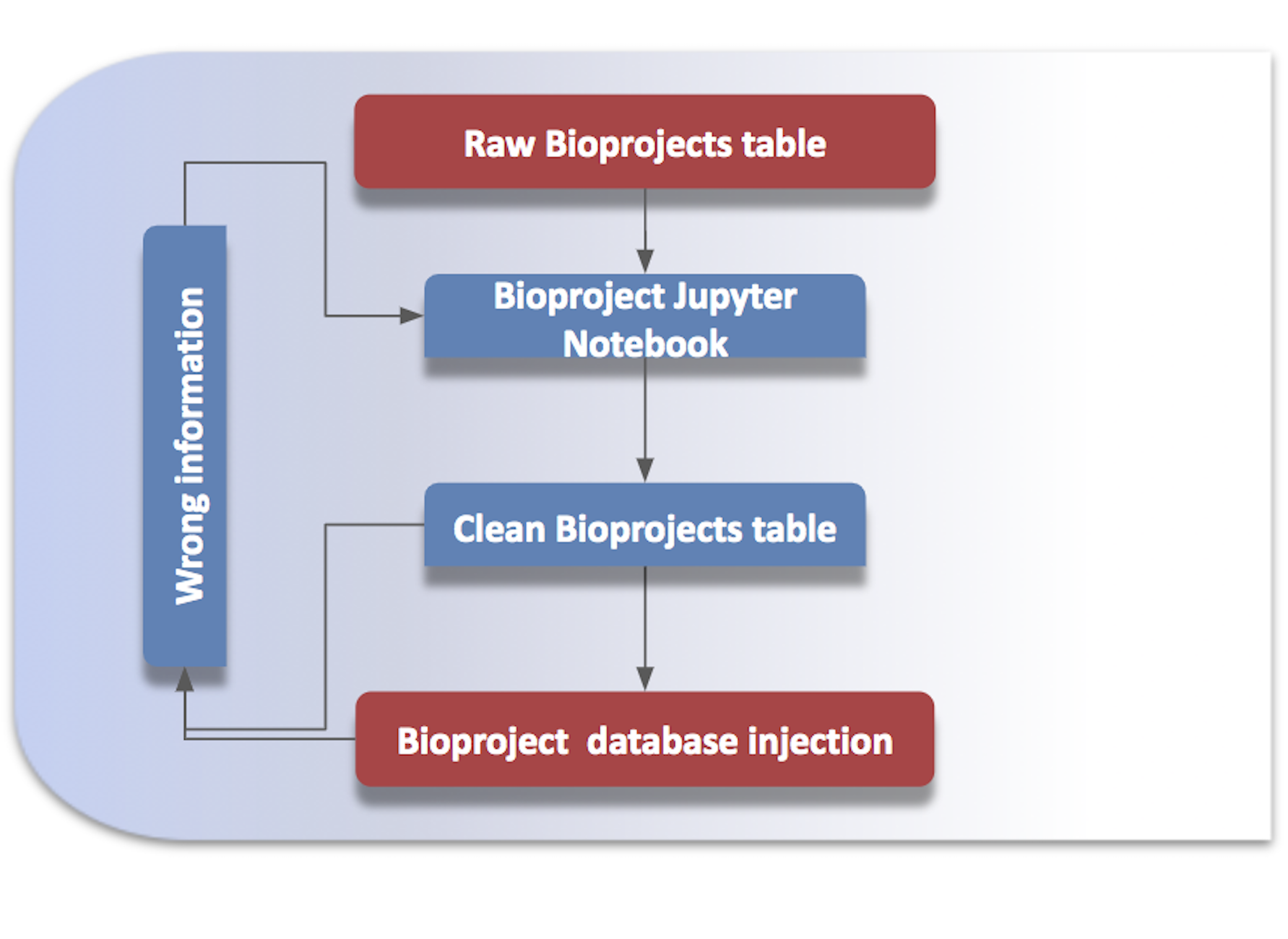

Supplement: baad092_Supp [file baad092_supp.zip › suppl_data/Supplementary_figure1_300dpi.png]

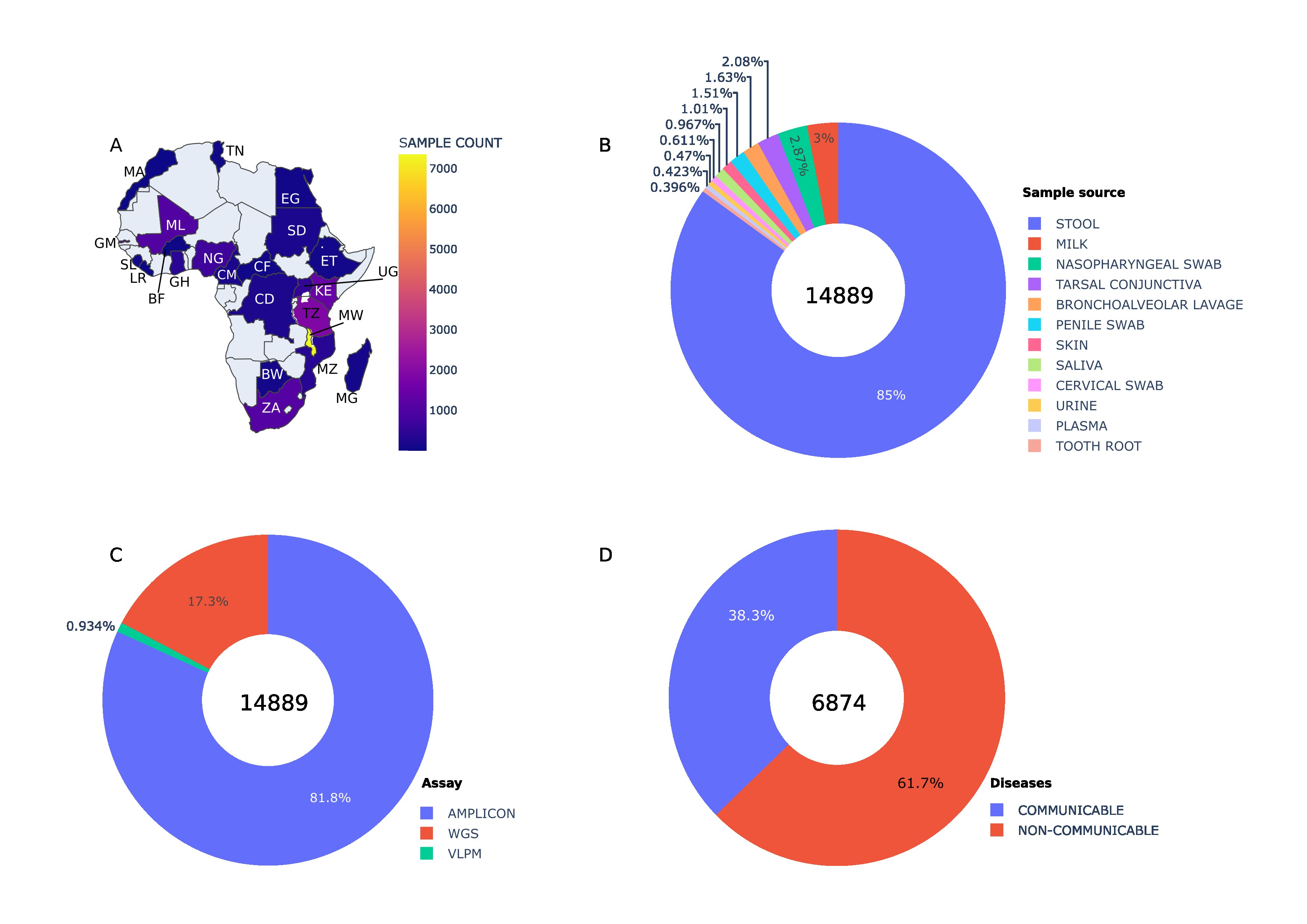

Supplement: baad092_Supp [file baad092_supp.zip › suppl_data/Supplementary_Figure2_300dpi.png]
